# Frequency and distribution patterns of opportunistic infections associated with HIV/AIDS in Uganda

**DOI:** 10.1186/s13104-016-2317-7

**Published:** 2016-12-07

**Authors:** John Rubaihayo, Nazarius M. Tumwesigye, Joseph Konde-Lule, Henry Wamani, Edith Nakku-Joloba, Fredrick Makumbi

**Affiliations:** 1 Department of Epidemiology and Biostatistics, School of Public Health, College of Health Sciences, Makerere University, Kampala, Uganda; 2 Department of Public Health, School of Health Sciences, Mountains of the Moon University, P.O. Box 837, Fort Portal, Uganda

**Keywords:** HIV/AIDS, Opportunistic infections, Antiretroviral therapy, Prevalence, TASO, Uganda

## Abstract

**Background:**

We conducted a study to assess the frequency and distribution patterns of selected opportunistic infections (OIs) and opportunistic cancers (OCs) in different geographical areas before and after HAART in Uganda.

**Methods:**

This was a cross-sectional serial review of observation data for adult HIV positive patients (≥15 years) enrolled with the AIDS support organization (TASO) in Uganda covering the period from January 2001 to December 2013. Both AIDS defining OIs/OCs and non-AIDS defining OIs were analyzed. The study period was structured into three time periods: “pre- HAART” (2001–2003), “early-HAART” (2004–2008) and “late-HAART” (2009–2013). Descriptive statistics were used to summarize the data by time period, age, gender and geographical location. Chi squared test used to test the significance of the differences in proportions.

**Results:**

A total of 108,619 HIV positive patients were included in the analysis. 64% (64,240) were female with median age of 33 years (IQR 27–40). The most frequent OIs before HAART were oral candida (34.6%) diarrhoeal infection (<1 month) (30.6%), geohelminths (26.5%), *Mycobacterium tuberculosis* (TB) (17.7%), malaria (15.1%) and bacterial pneumonia (11.2%). In early HAART (2004–2008), the most frequent OIs were geohelminths (32.4%), diarrhoeal infection (25.6%), TB (18.2%) and oral candida (18.1%). In late HAART (2009–2013), the most frequent OIs were geohelminths (23.5%) and diarrhoeal infection (14.3%). By gender, prevalence was consistently higher in women (p < 0.05) before and after HAART for geohelminths, candidiasis, diarrhoeal infection, bacterial pneumonia and genital ulcer disease but consistently higher in men for TB and Kaposi’s sarcoma (p < 0.05). By age, prevalence was consistently higher in older age groups (>30 years) before and after HAART for oral candida and TB (p < 0.05) and higher in young age groups (<30 years) for malaria and genital ulcers (p < 0.05). By geographical location, prevalence was consistently higher in Eastern and Northern Uganda before and after HAART for diarrheal infection and geohelminths (p < 0.0001).

**Conclusions:**

The frequency and pattern of OIs before and after HAART differs by gender, age and geographical location. Prevalence of geohelminths and diarrhea infection(<1 month) remains high especially in Northern and Eastern Uganda even after HAART and should therefore be given special attention in HIV/AIDS care programmes in these settings.

## Background

The human immunodeficiency virus (HIV) epidemic remains one of the greatest global health challenge of the 21st century [1] in the absence of an effective vaccine or curative therapy. According to the Joint United Nations Programme on HIV/AIDS (UNAIDS), 36.7 million people worldwide were estimated to be living with this deadly virus by end of 2015 of which 25.5 million (69.5%) were in sub-Saharan Africa [1]. Since the outbreak of the HIV pandemic, an estimated 34 million people worldwide have died and sub-Saharan Africa accounts for almost 70% of the total deaths [2]. However, with increased access to highly active antiretroviral therapy (HAART), there has been tremendous improvement in survival and quality of life among persons living with HIV globally. Latest UNAIDS data shows by end of 2015, close to 17 million persons living with HIV globally were on HAART with subsequent reduction in mortality of 43% [1].

Opportunistic infections (OIs) associated with HIV remain the single main cause of ill-health and death among HIV/AIDS patients in resource poor settings [3–5]. OIs lower the quality of life of HIV infected persons, speeds up the rate of progression to fully blown AIDS, reduces patients’ response to antiretroviral treatment especially when HIV-positive patients are co-infected with tuberculosis, increases stigma and limits one’s ability to work and are usually associated with high medical care costs [6, 7]. OIs have therefore greatly contributed to poverty among those infected and affected by HIV/AIDS and hence an impediment to the attainment of the sustainable development goal (SDG) three on health in resource poor settings.

Although the natural history of HIV tends to be similar in most patients, the patterns of OIs that largely define the symptomatic and clinical manifestation of HIV infection tend to vary in different regions of the world [4, 8, 9]. Thus, while HIV patients in developed countries rarely suffer from bacterial and protozoal infections, they are a major cause of morbidity and mortality in resource-poor settings [4, 9, 10]. Though, the introduction of highly active antiretroviral therapy (HAART) has substantially reduced the risk of suffering from an opportunistic infection [11], HIV positive patients in resource poor settings continue to suffer from opportunistic infections due to several factors including late HIV diagnosis, sub-optimal HAART use, poor adherence, dug resistance, poverty, poor nutrition, high exposure to infectious agents, just to mention a few [10, 12–17].

Uganda is one of the few sub-Saharan countries in which the magnitude of the HIV epidemic has been substantially reduced and stabilized in the past decades; though recent reports show a slight increase in HIV prevalence among adults from a national average of 6.4% in 2005 to 7.3% in 2011 [18]. According to the Uganda AIDS indicator survey (2011), the burden of HIV varies by person (gender and age) and geographical area being predominantly higher in women (8.3%) compared to men (6.1%). By age, prevalence was found higher in older age groups (>35 years) [18]. Geographically, the central region of Uganda was shown to have the highest HIV prevalence (10.6%), followed by mid-northern (8.3%) and then mid and south western (8–8.2%) and lowest prevalence in mid-eastern (4.1%) [18]. However, what was not clear was whether the burden of HIV related opportunistic infections follows the same pattern. The role of different OIs in morbidity before and after HAART has never been well documented. The purpose of this study was to assess the frequency, distribution patterns of different OIs before and after HAART in Uganda.

## Methods

### Study setting

The study obtained observational data from the AIDS support organization (TASO) known to be the oldest and largest HIV/AIDS care and treatment program in Uganda and sub-Saharan Africa. TASO was founded in 1987 and has 11 HIV/AIDS clinics spread across Uganda which have been nationally recognized as centres of excellence (CoE) in HIV/AIDS care and treatment in Uganda. TASO HIV clinics offer comprehensive HIV treatment and care, including provision of free antiretroviral drugs and cotrimoxazole prophylaxis, HIV testing and counselling, home-based care and psycho-social support to their clients. TASO HAART programme started as part of the National HAART roll-out programme in public health facilities in Uganda. Being one of the largest HAART providers in the country, TASO attracted a lot of support from different funders supporting HAART programmes in sub-Saharan Africa including the President’s Emergency Plan for AIDS Relief (PEPFAR) and the Global Fund to Fight AIDS, Tuberculosis and Malaria. Initially, HAART eligibility was based on WHO 2006 guidelines i.e. WHO stage 3 or 4 illness or a CD4 cell count <200 cells/μl for adults and adolescents and WHO stage III, advanced stage II or stage I with CD4 cell percentage less than 20% for those more than 18 months of age [20]. However, in 2010 new HAART guidelines [21] that raised the threshold for adults and adolescents to a CD4 cell count ≤350 or WHO clinical stage 3 or 4 irrespective of CD4 cell count were adopted [22]. Those not eligible for HAART were offered cotrimoxazole or dapsone prophylaxis. Additionally TASO HAART delivery includes community volunteers/treatment partners that help to monitor HAART adherence, adverse effects, opportunistic infections and reporting those who die. All services are free of charge including anti-retroviral drugs (ARVs) for those who are eligible [23].

### Study design

This was a serial cross-sectional review of observation data for adult HIV positive patients (≥15 years) obtaining care and treatment from the AIDS support organization (TASO) in Uganda covering the period from 1st January 2001 to 31st December 2013. A total of 17 opportunistic infections including 14 AIDS-defining opportunistic infections (*Oral candida, Esophageal candida, Mycobacterium tuberculosis, Genital ulcer disease, Cryptococcal meningitis, Herpes zoster, Bacterial pneumonia, Diarrhoeal infection* <1 month, *Cryptosporidiosis, Herpes simplex labialis, Cytomegalovirus, Toxoplasmosis, Pneumocystis jiroveci pneumonia, Oral hairy leukoplakia*), one opportunistic cancer (*Kaposi’s sarcoma*) and three non-AIDS defining opportunistic infections (*Malaria and Geoheminths*) were the main focus of this study. The study time was structured into three time periods corresponding to two important milestones in HIV care and treatment in Uganda. The first time period was designated as “pre-HAART” (2001–2003) when HAART was not available. The second time period was designated as “early HAART” (2004–2008) when HAART access was limited to only severely ill patients (CD4 count ≤200 cells/µl regardless of clinical stage or WHO stage III or IV disease) [19, 20]. The third time period was designated as “late HAART” (2009–2013) when HAART access was expanded to include patients with CD4 cell count >200 cells/µl but ≤350 cells/µl or had WHO stage III or IV disease regardless of CD4 cell count [21].

### Sampling and sample size

**Fig. 1:**
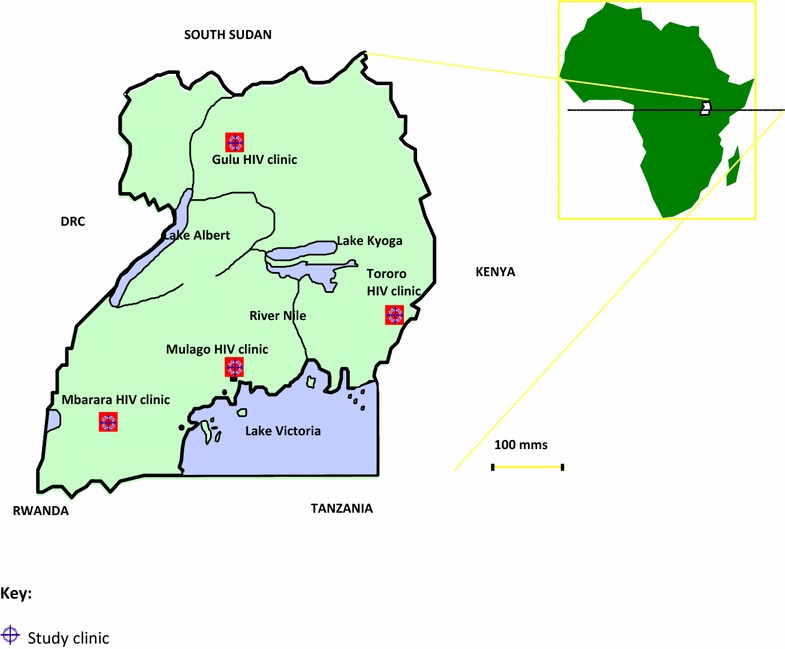
Map of Uganda showing geographical location of study sites

Four TASO HIV clinics were purposively selected basing on volume and quality of data available and geographical representation. The HIV clinics selected were TASO Mulago HIV clinic in central Uganda, TASO Mbarara HIV clinic in south-western Uganda, TASO Tororo HIV clinic in Eastern Uganda and TASO Gulu HIV clinic in Northern Uganda (Fig. 1). All HIV positive adults (15 years and above) who attended at least once at the selected HIV clinics in the period from 1st January 2001 to 31st December 2013 were included in the study.

### Data collection

TASO medical staff systematically registered the clients’ demographic characteristics and medical information following an established protocol for all TASO HIV clinics. In brief, clients were expected to attend the clinic at least once a month. At each clinic visit, data per client was collected on a standardized case report form (CRF) detailing the client’s demographic information, clinical condition, medical history, OI diagnosis, ART use, prophylaxis use, any other treatment given and side effects/toxicities if any. OI diagnosis was based on WHO and Uganda ministry of Health guidelines [20–22, 24]. Data were then compiled and entered into the TASO electronic data base by TASO data administrator using EPIINFO vs3 in Access format. Monthly medical data for each participant covering the period January 2001 to December 2013 were extracted by the data administrator, delinked from overt identifiers and then handed over for analysis.

### Data analysis

The extracted data were analyzed using Stata statistical software version 13.1 (Stata Corp, College station, Texas, USA). Descriptive statistics were summarized by frequencies and percentages. For categorical variables, Chi squared test was used to test for the differences in proportions and Wilcoxon rank-sum test for metric variables. Prevalence was calculated from the number patients ever diagnosed with a particular OI divided by the total number of patients who attended monthly clinic visits at least once in a given period. All significance tests were two sided with a p value <0.05 considered significant.

## Results

### Socio-demographic and baseline clinical characteristics

A total of 108,619 HIV positive individuals were included in the analysis of which 64% were female with median age of 33 years (IQR 27–40). Majority were subsistence farmers (83%) with primary or no education (77%), largest number were married (47%) and were Catholics by religion/faith (45%) (Table 1). Gulu HIV clinic in Northern Uganda had the largest number of Catholics (73%) While Mbarara HIV clinic in Western Uganda had the largest number of Protestants (51%). Tororo HIV clinic in Eastern Uganda had the largest number of marrieds (54%). Mulago HIV clinic in Central Uganda had the largest number of divorcees (27%), highly educated (>secondary) (34%) and fewer subsistence farmers (18%).

**Table 1:** Demographic characteristics of the study participants, total and clinic-specific

Variable	Total number n (%)	Tororo HIV clinic n (%)	Mulago HIV clinic n (%)	Mbarara HIV clinic n (%)	Gulu HIV clinic n (%)
Gender (n = 108,619)					
Female	69,240 (64)	16,458 (62)	21,498 (67)	19,350 (63)	11,934 (62)
Male	39,379 (36)	9966 (38)	10,803 (33)	11,433 (37)	7177 (38)
Median age (IQR) (n = 107,112)	33 (27, 40)	34 (28, 41)	33 (27, 40)	33 (27, 40)	33 (26, 40)
Occupation (n = 86,136)					
Paid employee	15,061 (17)	2337 (11)	6613 (27)	2929 (13)	3182 (18)
Self employed	31,073 (36)	6905 (32)	10,975 (46)	5516 (24)	7677 (43)
Subsistence farmer	34,564 (40)	11,250 (52)	4390 (18)	12,635 (56)	6289 (35)
Others	5438 (6)	1105 (5)	2075 (9)	1485 (7)	773 (4)
Education (n = 92,840)					
None	19,712 (21)	6009 (28)	2672 (11)	6258 (21)	4773 (27)
Primary	51,768 (56)	11,768 (54)	13,136 (55)	17,046 (58)	9818 (55)
Secondary	17,908 (19)	3236 (15)	6831 (28)	5102 (17)	2739 (15)
Tertiary or above	3452 (4)	640 (3)	1376 (6)	855 (3)	581 (3)
Marital status (n = 93,004)					
Single/never married	5747 (6)	1587 (7)	1652 (7)	1816 (6)	692 (4)
Married/cohabiting	43,661 (47)	11,600 (54)	10,203 (42)	13,369 (45)	8489 (47)
Divorced	17,138 (18)	2783 (13)	6409 (27)	4802 (16)	3144 (17)
Widowed	21,698 (23)	4923 (23)	4739 (20)	7535 (26)	4501 (25)
Others	4760 (5)	701 (3)	1034 (4)	1897 (6)	1128 (6)
Religion/faith (n = 91,826)					
Catholic	41,772 (45)	9169 (42)	9022 (38)	10,604 (37)	12,977 (73)
Protestant	33,171 (36)	7166 (33)	8290 (34)	14,642 (51)	3073 (17)
Muslim	7114 (8)	1740 (8)	3288 (14)	1627 (6)	459 (3)
Pentecostal	6835 (7)	2440 (11)	2536 (11)	874 (3)	985 (5)
Others	2934 (3)	1078 (5)	840 (3)	698 (2)	318 (2)

### Frequency and distribution patterns of OIs

Opportunistic infections (OIs) accounted for 99% of morbidity causes compared to 1% due to opportunistic cancers (Kaposi’s sarcoma, Burkitti’s lymphoma and Malignant melanoma). Overall, from 2001 to 2013, a total of 291,168 OI episodes were recorded mainly caused by 16 opportunistic infections and Kaposi’s sarcoma which though not an opportunistic infection but was included because of its infectious cause (human herpes virus type 8) [25]. Overall the burden of OIs was largely due to geohelminths (30.7%), diarrhoeal infection <1 month (25.5%), oral candida (19.4%), *M. tuberculosis* (18.3%), bacterial pneumonia (14.8%) and genital ulcers (10%), others were below 10% (Table 2).

**Table 2:** Overall frequency distribution of different types of OIs among HIV positive patients in TASO, Uganda (2001–2013)

	Overall (2001–2013)	
	Frequency (n = 108,619)	Percent^a^
Opportunistic infection		
Geo helminthes	33,311	30.7
Diarrhea <1 month	27,658	25.5
Oral candida	21,053	19.4
*Mycobacterium tuberculosis*	19,825	18.3
Bacterial pneumonia	16,076	14.8
Genital ulcer	10,887	10.0
Confirmed malaria	8924	8.2
Esophageal candida	8778	8.0
Herpes zoster	7113	6.5
Cryptosporidiosis	3551	3.3
Cryptococcosis	1725	1.6
Herpes simplex labialis	1367	1.3
Cytomegalovirus	616	0.6
Toxoplasmosis	589	0.5
*Pneumocystis carini/jiroveci pneumonia*	374	0.3
Oral hairy leukoplakia	360	0.3
Hepatitis B or C virus	303	0.3
Opportunistic cancer		
Kaposis sarcoma/HHV8	1155	1.1

*HHV8* human herpes virus type 8, *n* number of patients

^a^Proportion of study participants ever diagnosed with a particular OI in the period 2001–2013

**Fig. 2:**
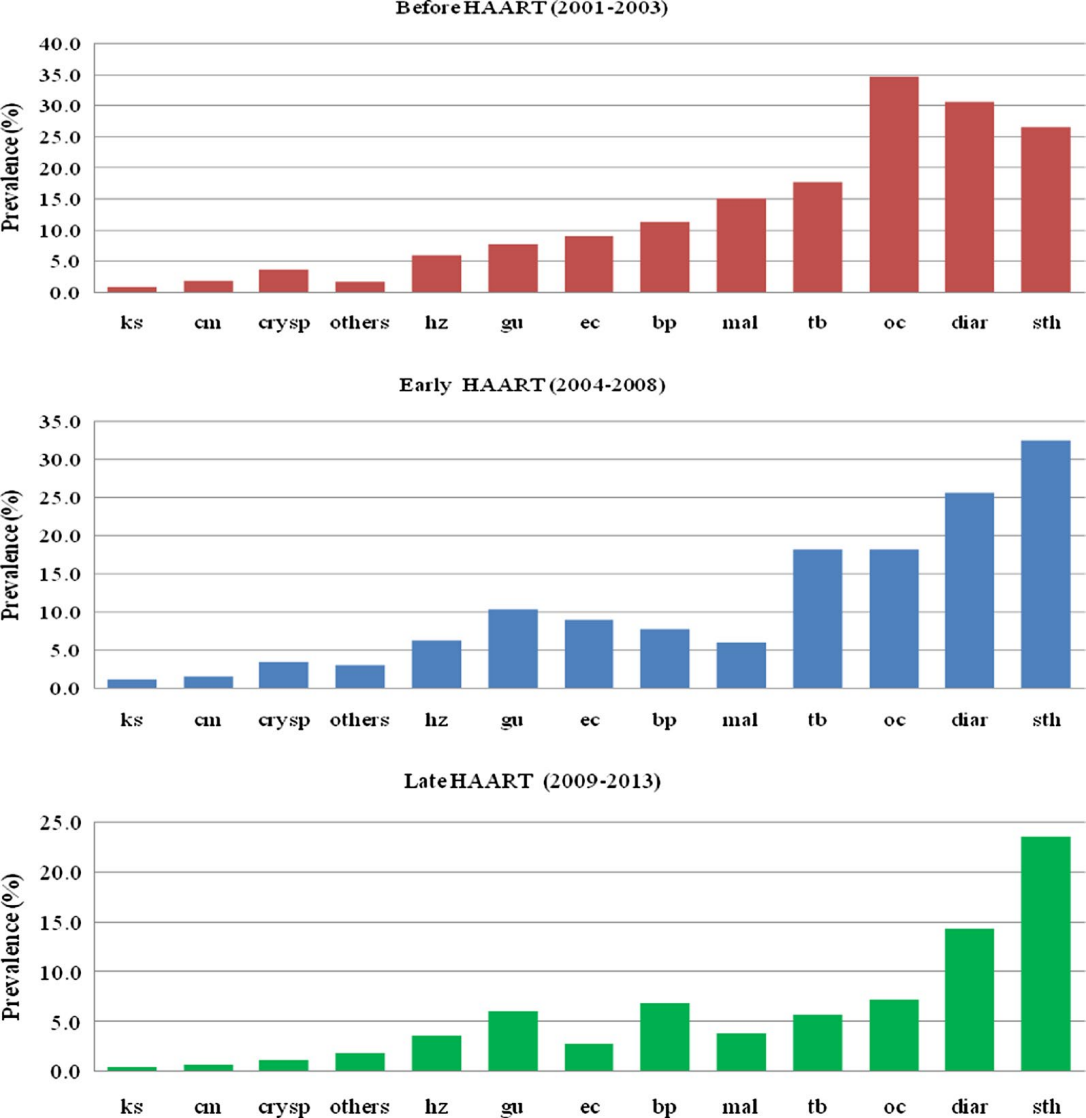
Bar charts showing period prevalence of selected OIs among HIV positive patients in TASO, Uganda before HAART, early and late HAART. *sth* soil transmitted helminthes, *diar* diarrhea, *cm* cryptococcal meningitis, *ec* esophageal candida, *tb* tuberculosis, *oc* oral candida, *bp* bacterial pneumonia, *gu* genital ulcer, *ks* Kaposis sarcoma, *crysp* cryptosporidiosis, *hz* herpes zoster, *mal* malaria, others (*Herpes simplex labialis*, *Toxoplasmosis*, *Cytomegalovirus*, *Pneumocystis carinii/jiroveci pneumonia*)

Before HAART (2001–2003), the most frequent OIs were oral candida (34.6%) diarrhoea <1 month (30.6%), geohelminths (26.5%), *M. tuberculosis* (17.7%), malaria (15.1%) and bacterial pneumonia (11.2%), the rest were below 10%. In early HAART (2004–2008), the most frequent OIS (>10%) were geohelminths (32.4%), diarrhoea <1 month (25.6%), *M. tuberculosis* (18.2%) and oral candida (18.1%). In late HAART (2009–2013), the most frequent OIs (>10%) were geohelminths (23.5%) and diarrhoea <1 month (14.3%) (Fig. 2).

By gender, prevalence was greater in women for most OIs except for *M. tuberculosis* and Kaposi’s sarcoma (Table 3). By age, prevalence was generally higher in older age groups (>30 years) for most OIs except malaria and genital ulcers which were predominantly higher in younger age groups [<30 years (Table 4)].

**Table 3:** Chi squared test for the difference in OI prevalence by gender before and after HAART

	Prevalence		p value
	Women (n = 16,519), n (%)	Men (n = 7848), n (%)	
Before HAART (2001–2003)			
*Opportunistic infection*			
Oral candida	5987 (36.2)	2449 (31.2)	<0.0001
Diarrhoea	5164 (31.3)	2295 (29.2)	<0.0001
Geohelminths	4544 (27.5)	1912 (24.4)	<0.0001
*Mycobacterium tuberculosis*	2769 (16.8)	1544 (19.7)	<0.0001
Confirmed malaria	2582 (15.6)	1087 (13.9)	<0.0001
Bacterial pneumonia	1928 (11.7)	809 (10.3)	<0.0001
Esoph candida	1620 (9.8)	567 (7.2)	<0.0001
Genital ulcer	1430 (8.7)	433 (5.5)	<0.0001
Herpes zoster	1011 (6.1)	418 (5.3)	0.025
Cryptosporidiosis	624 (3.8)	271 (3.5)	0.076
Cryptococcosis	275 (1.7)	152 (1.9)	0.702
Others	301 (1.8)	90 (1.1)	0.019
*Opportunistic cancer*			
Kaposi’s sarcoma/HHV8	114 (0.7)	95 (1.2)	0.068

	Prevalence		p value
	Women (n = 38,599), n (%)	Men (n = 20,232), n (%)	
Early HAART (2004–2008)			
*Opportunistic infection*			
Geohelminths	13,205 (34.2)	5858 (29.0)	<0.0001
Diarrhoea	10,231 (26.5)	4812 (23.8)	<0.0001
Oral candida	7710 (20.0)	2961 (14.6)	<0.0001
*Mycobacterium tuberculosis*	3419 (16.9)	7262 (18.8)	<0.0001
Bacterial pneumonia	1741 (8.6)	2839 (7.4)	<0.0001
Genital ulcer	4461 (11.6)	1660 (8.2)	<0.0001
Esoph candida	3872 (10.0)	1456 (7.2)	<0.0001
Confirmed malaria	2482 (6.4)	1111 (5.5)	0.023
Herpes zoster	2606 (6.8)	1152 (5.7)	0.037
Cryptosporidiosis	1373 (3.6)	662 (3.3)	0.061
Cryptococcosis	602 (1.6)	332 (1.6)	0.097
Others	1260 (3.3)	539 (2.7)	0.06
*Opportunistic cancer*			
Kaposi’s sarcoma/HHV8	347 (0.9)	330 (1.6)	0.012

	Prevalence		p value
	Women (n = 40,335), n (%)	Men (n = 22,547), n (%)	
Late HAART (2009–2011)			
*Opportunistic infection*			
Geohelminths	9951 (24.7)	4852 (21.5)	<0.0001
Diarrhoea	6056 (15.0)	2917 (12.9)	<0.0001
Oral candida	3063 (7.6)	1446 (6.4)	0.029
Bacterial pneumonia	2895 (7.2)	1453 (6.4)	0.045
Genital ulcer	2581 (6.4)	1202 (5.3)	0.017
*Mycobacterium tuberculosis*	2101 (5.2)	1489 (6.6)	0.024
Confirmed malaria	1595 (4.0)	789 (3.5)	0.540
Herpes zoster	1522 (3.8)	748 (3.3)	0.490
Esoph candida	1163 (2.9)	594 (2.6)	0.046
Cryptosporidiosis	464 (1.2)	231 (1.0)	0.792
Cryptococcosis	251 (0.6)	166 (0.7)	0.09
Others	759 (1.9)	390 (1.7)	0.102
*Opportunistic cancer*			
Kaposi’s sarcoma/HHV8	150 (0.4)	160 (0.7)	0.015

*HHV8* human herpes virus 8, *n* number of patients

**Table 4:** Chi squared test for the difference in OI prevalence by age at enrolment before and after HAART

	Prevalence			p value
	Age < 30 (n = 8070), n (%)	Age = 30–39 (n = 10,008), n (%)	Age ≥ 40 (n = 6223), n (%)	
Before HAART (2001–2003)				
*Opportunistic infection*				
Geohelminths	2187 (27.1)	2536 (25.3)	1714 (27.5)	0.058
Diarrhoea	2422 (30.0)	3117 (31.1)	1907 (30.6)	0.671
Oral candida	2586 (32.0)	3625 (36.2)	2213 (35.6)	<0.001
*Mycobacterium tuberculosis*	1350 (16.7)	1706 (17.0)	1251 (20.1)	0.031
Bacterial pneumonia	905 (11.2)	1113 (11.1)	706 (11.3)	0.139
Genital ulcer	667 (8.3)	802 (8.0)	390 (6.3)	0.026
Confirmed malaria	1277 (15.8)	1518 (15.2)	863 (13.9)	0.029
Esoph candida	654 (8.1)	945 (9.4)	585 (9.4)	0.126
Herpes zoster	505 (6.3)	568 (5.7)	351 (5.6)	0.198
Cryptosporidiosis	269 (1.6)	387 (1.7)	238 (1.6)	0.218
Cryptococcosis	133 (1.6)	182 (1.8	110 (1.8)	0.707
Others	130 (3.3)	175 (3.9)	99 (3.8)	0.168
*Opportunistic cancer*				
Kaposi’s sarcoma/HHV8	57 (0.7)	104 (1.0)	48 (0.8)	0.052

	Prevalence			p value
	Age < 30 (n = 17,872), n (%)	Age = 30-39 (n = 23,395), n (%)	Age ≥ 40 (n = 16,935), n (%)	
Early HAART (2004–2008)				
*Opportunistic infection*				
Geohelminths	5849 (32.7)	7690 (32.9)	5645 (33.3)	0.365
Diarrhoea	4323 (24.2)	6034 (25.8)	4555 (26.9)	0.010
Oral candida	2919 (16.3)	4510 (19.3)	3191 (18.8)	<0.0001
*Mycobacterium tuberculosis*	3073 (17.2)	4366 (18.7)	3150 (18.6)	0.006
Bacterial pneumonia	1277 (7.1)	1930 (8.2)	1331 (7.9)	0.009
Genital ulcer	1850 (10.4)	2634 (11.3)	1609 (9.5)	<0.0001
Confirmed malaria	1230 (6.9)	1366 (5.8)	883 (5.2)	<0.0001
Esoph candida	1342 (7.5)	2309 (9.9)	1648 (9.7)	<0.0001
Herpes zoster	1080 (6.0)	1554 (6.6)	1106 (6.5)	0.407
Cryptosporidiosis	554 (2.6)	823 (3.0)	649 (3.6)	0.039
Cryptococcosis	236 (1.3)	399 (1.7)	286 (1.7)	0.011
Others	464 (3.1)	712 (3.5)	608 (3.8)	0.07
*Opportunistic cancer*				
Kaposi’s sarcoma/HHV8	166 (0.9)	290 (1.2)	221 (1.3)	0.009

	Prevalence			p value
	Age < 30 (n = 21,479), n (%)	Age = 30-39 (n = 23,168), n (%)	Age ≥ 40 (n = 17,174), n (%)	
Late HAART (2009–2013)				
*Opportunistic infection*				
Geohelminths	5230 (24.3)	5489 (23.7)	4119 (24.0)	0.598
Diarrhoea	3153 (14.7)	3352 (14.5)	2372 (13.8)	0.025
Oral candida	1442 (6.7)	1748 (7.5)	1305 (7.6)	0.015
*Mycobacterium tuberculosis*	1194 (5.6)	1423 (6.1)	911 (5.3)	0.008
Bacterial pneumonia	1481 (6.9)	1601 (6.9)	1218 (7.1)	0.673
Genital ulcer	1454 (6.8)	1479 (6.4)	827 (4.8)	<0.0001
Confirmed malaria	934 (4.3)	896 (3.9)	524 (3.1)	<0.0001
Esoph candida	595 (2.8)	662 (2.9)	492 (2.9)	0.958
Herpes zoster	775 (3.6)	856 (3.7)	670 (3.9)	0.069
Cryptosporidiosis	228 (1.7)	259 (1.7)	204 (2.2)	0.027
Cryptococcosis	113 (0.5)	178 (0.8)	124 (0.7)	0.010
Others	362 (1.1	405 (1.1)	374 (1.2)	0.973
*Opportunistic cancer*				
Kaposi’s sarcoma/HHV8	86 (0.4)	132 (0.6)	91 (0.5)	0.055

*HHV8* human herpes virus 8, *n* number of patients

By geographical location, highest prevalence before HAART was observed in Tororo HIV clinic in Eastern Uganda for diarrhea <1 month (43.1%), geohelminths (40.9%) and TB (12.7%); Mbarara HIV clinic in South-western Uganda for oral candida (43.1%) and malaria (16.7); Mulago HIV clinic in Central Uganda for genital ulcers (12.6%). In Early HAART, highest prevalence was observed: in Gulu HIV clinic in Northern Uganda for geohelminths (37.7%), diarrhea <1 month (36.8%), TB (21.0%) and malaria (10.6%); in Mbarara HIV clinic in South-western Uganda for oral candida (24%) and Mulago HIV clinic in Central Uganda for genital ulcers (12.6%). In late HAART, highest prevalence was observed in Gulu HIV clinic in Northern Uganda for geohelminths (26.3%) and Tororo HIV clinic in Eastern Uganda for diarrhea <1 month (19.0%) (Table 5).

**Table 5:** Chi squared test for the difference in OI prevalence by geographical location

	Prevalence			p value
	Tororo (Eastern Uganda) (n = 6534), n (%)	Mulago (Central Uganda) (n = 7243), n (%)	Mbarara (South-western Uganda) (n = 10,586), n (%)	
Before HAART (2001–2003)				
*Opportunistic infection*				
Geohelminths	2671 (40.9)	1406 (19.4)	2378 (22.5)	<0.0001
Diarrhoea	2817 (43.1)	1780 (24.6)	2862 (27.0)	<0.0001
Oral candida	1531 (23.4)	2345 (32.4)	4560 (43.1)	<0.0001
Bacterial pneumonia	858 (13.1)	896 (12.4)	983 (9.3)	<0.0001
*Mycobacterium tuberculosis*	830 (12.7)	875 (12.1)	608 (5.7)	<0.0001
Genital ulcer	508 (7.8)	697 (9.6)	658 (6.2)	<0.0001
Confirmed malaria	1072 (16.4)	826 (11.4)	1771 (16.7)	<0.0001
Esoph candida	261 (4.0)	732 (10.1)	1194 (11.3)	<0.0001
Herpes zoster	388 (5.9)	423 (5.8)	618 (5.8)	0.098
Cryptosporidiosis	178 (2.7)	283 (3.9)	434 (4.1)	<0.0001
Cryptococcosis	160 (2.4)	145 (2.0)	122 (1.2)	<0.0001
Others	75 (1.1)	204 (2.8)	112 (1.1)	<0.0001
*Opportunistic cancer*				
Kaposi’s sarcoma/HHV8	96 (1.5)	61 (0.8)	52 (0.5)	<0.0001

	Prevalence				p value
	Tororo (Eastern Uganda) (n = 16,207), n (%)	Mulago (Central Uganda) (n = 16,288), n (%)	Mbarara (South western Uganda) (n = 17,047), n (%)	Gulu (Northern Uganda) (n = 10,464), n (%)	
Early HAART (2004–2008)					
*Opportunistic infection*					
Geohelminths	5825 (35.9)	4363 (26.8)	4925 (28.9)	3950 (37.7)	<0.0001
Diarrhoea	4503 (27.8)	3261 (20.0)	3431 (20.1)	3848 (36.8)	<0.0001
Oral candida	1966 (12.1)	3632 (22.3)	4097 (24.0)	976 (9.3)	<0.0001
*Mycobacterium tuberculosis*	2958 (18.2)	3589 (22.1)	1936 (11.4)	2198 (21.0)	<0.0001
Bacterial pneumonia	1301 (8.0)	1627 (10.0)	823 (4.8)	829 (7.9)	<0.0001
Genital ulcer	1373 (8.5)	2046 (12.6)	1474 (8.6)	1229 (11.7)	<0.0001
Confirmed malaria	893 (5.5)	984 (6.0)	611 (3.6)	1105 (10.6)	<0.0001
Esoph candida	969 (6.0)	1744 (10.7)	1714 (10.1)	901 (8.6)	<0.0001
Herpes zoster	1103 (6.8)	1110 (6.8)	970 (5.7)	575 (5.5)	<0.0001
Cryptosporidiosis	666 (4.1)	467 (2.9)	419 (2.5)	483 (4.6)	<0.0001
Cryptococcosis	238 (1.5)	337 (2.1)	197 (1.2)	162 (1.5)	<0.0001
Others	364 (2.2)	647 (4.0)	333 (2.0)	455 (4.3)	<0.0001
*Opportunistic cancer*					
Kaposi’s sarcoma/HHV8	209 (1.3)	195 (1.2)	128 (0.8)	145 (1.4)	0.003

	Prevalence				p value
	Tororo (Eastern Uganda) (n = 14,658), n (%)	Mulago (Central Uganda) (n = 17,977), n (%)	Mbarara (South western Uganda) (15,095), n (%)	Gulu (Northern Uganda) (n = 15,152), n (%)	
Late HAART (2009–2013)					
*Opportunistic infection*					
Geohelminths	3270 (22.3)	3373 (18.8)	3169 (21.0)	3991 (26.3)	<0.0001
Diarrhoea	2786 (19.0)	1949 (10.8)	1653 (11.0)	2585 (17.1)	<0.0001
Oral candida	731 (5.0)	1822 (10.1)	1617 (10.7)	339 (2.2)	<0.0001
Bacterial pneumonia	1354 (9.2)	1043 (5.8)	641 (4.2)	1310 (8.6)	<0.0001
*Mycobacterium tuberculosis*	780 (5.3)	1088 (6.1)	436 (2.9)	1286 (8.5)	<0.0001
Genital ulcer	767 (5.2)	1047 (5.8)	1265 (8.4)	704 (4.6)	<0.0001
Confirmed malaria	769 (5.2)	688 (3.8)	365 (2.4)	562 (3.7)	<0.0001
Esoph candida	342 (2.3)	640 (3.6)	489 (3.2)	286 (1.9)	<0.0001
Herpes zoster	581 (4.0)	739 (4.1)	554 (3.7)	396 (2.6)	<0.0001
Cryptosporidiosis	219 (1.5)	198 (1.1)	140 (0.9)	138 (0.9)	0.011
Cryptococcosis	198 (1.4)	96 (0.5)	56 (0.4)	67 (0.4)	<0.0001
Others	356 (2.4)	351 (2.0)	239 (1.6)	203 (1.3)	<0.0001
*Opportunistic cancer*					
Kaposi’s sarcoma/HHV8	116 (0.8)	91 (0.5)	58 (0.4)	45 (0.3)	<0.0001

*HHV8* human herpes virus 8, *n* number of patient

## Discussion

The study summaries clinical data representing nearly 7% (108,619/1.6 million) of Ugandans living with HIV/AIDS. Majority were women (64%), with low education (primary or none) (77%) and of very low socio-economic status (venders/petty traders/subsistence farmers) (76%). This nature of patients is comparable to other HIV positive patients found elsewhere in ART programmes in sub-Saharan Africa [17, 26–28]. A study in Nigeria, in which both pre-HAART and post-HAART data for HIV positive patients in care were analyzed, 65.8% were found to be women and more than half (50.4%) were of low socio-economic status [28]. Another study that examined data from an ART cohort in a rural hospital in western Uganda also found 65.3% of the beneficiaries were women and 77.4% were of low socio-economic class (unemployed/self-employed/subsistence farmers) [26]. The fact that there were twice as many women than men in the current study is additional evidence that men remain under-represented in most ART programmes in sub-Saharan Africa with consequently less favourable programme outcomes.

Overall, opportunistic infections (OIs) occurred most and accounted for 99% of all opportunistic events compared to 1% due to opportunistic cancers (Kaposi’s sarcoma, malignant melanomas, Burkitt’s lymphoma and other lymphomas). This is also additional evidence that opportunistic infections as opposed to opportunistic cancers are the primary cause of morbidity and mortality among HIV positive individuals in sub-Saharan Africa [6]. Histoplasmosis, *Mycobacterium avium complex* were not seen in the current study providing additional evidence that these OIs might be absent or there is lack of diagnostic capacity in sub-Saharan Africa [29, 30]. The apparent rarity of Cytomegalovirus, *Pneumocystis jiroveci pneumonia*, Cryptosporidiosis and Toxoplasmosis in the current study could also be due to lack of diagnostic capacity or evidence that they are rare among African HIV positive patients. The low prevalence of Cryptococcal meningitis (CM) and Kaposi’s sarcoma (KS) could perhaps be due to the fact that TASO HIV clinics being mainly out-patient clinics and CM and KS being referral conditions, cases may have opted to seek for specialized care elsewhere. However, even other studies elsewhere in sub-Saharan Africa have shown low prevalence of both CM and KS. For example a study in Nigeria found prevalence of CM and KS was relatively lower at 0.6 and 0.3% respectively compared to candidiasis (8.6%) and TB (7.7%) [28].

Before HAART (2001–2003) the burden of OIs, as expected, was indeed very high. The most common OIs before HAART were oral candida (34.6%), diarrhoeal <1 month (30.6%), geohelminths (26.5%), *M. tuberculosis* (17.7%), malaria (15.1%) and bacterial pneumonia (11.2%). This might be because they are highly endemic in the country and easy to diagnose. The most frequent OIs after HAART (late HAART) were diarrhoea <1 month and geohelminths. Similar OIs were also observation in other previous studies in African settings [17, 28, 31, 32].

In the current study, oral candidiasis was the most frequent opportunistic infection before HAART. According to Staine [6], oral candida caused by the fungus *Candida albicans* has been associated with HIV infection ever since HIV/AIDS was first reported in early 1980s [6]. In some studies in developed countries, the prevalence of oral candidiasis was as high as 44.8% in USA [9] and 23% in France [33] before HAART was introduced. A review of studies on HIV/AIDS related opportunistic infections in sub-Saharan Africa, showed the prevalence of oral candidiasis ranged from 14% in HIV infected pregnant women attending a rural hospital in Cameroon [34] to 67% among Senegalese in patients with AIDS [35] before HAART. However, most studies in the HAART era, show that it’s prevalence has reduced but is still common among HIV infected persons [15, 36–38]. In Uganda, most studies before HAART reported high prevalence of oral candidiasis among HIV-infected persons [39–41]. In the current study we have found that the prevalence of oral candida has substantially reduced though not completely eliminated probably because it is endemic in the country and common even among those who are not HIV positive. The risk for oral was higher in women and with old age but varied by geographical area being more common in Mbarara in South-western Uganda. The reasons for these geographical differences require further investigations.

In the current study, diarrhea <1 month was most frequent OI before and after HAART. Previous studies show up to 60% of people living with HIV experience diarrhoea, that negatively affects their quality of life and adherence to HAART [42]. Diarrhoea among HIV positive individuals may be due to multiple causes including infectious causes (bacterial, viral, protozoal, helminthic, etc.) or non-infectious causes(ARV drug effects e.g. ritonavir-boosted protease inhibitors such as lopinavir/ritonavir or nelfinavir) [42–46]. In Uganda, a previous study reported the commonest causes of diarrhoea to be helminthic infections (29.5%), bacterial infections (19.2%) and protozoal infections (9.2%) [47]. In the current study we found diarrhoea <1 months was more common among women compared to men probably because women by nature of their traditional responsibilities tend to be more vulnerable than men. Prevalence of diarrhea <1 month was found higher among HIV positive patients at Gulu HIV clinic in Northern and Tororo HIV clinic in Eastern Uganda compare to HIV positive patients at Mulago HIV clinic in Central Uganda and Mbarara HIV clinic in South-western Uganda probably because of the socio-economic disparities between these regions with the later being relatively more developed compared to the former. Though preventable, the persistence of diarrhoea <1 month after HAART and free access to universal anti-microbial prophylaxis needs further investigations to establish the actual cause.

TB caused by *M. tuberculosis* is one of the leading causes of morbidity and mortality in persons infected with HIV/AIDS globally [48]. Studies show that HIV reactivates latent TB hence increasing the risk of TB in HIV-infected patients [49]. In 2012, about 13% of the people who developed TB globally were HIV positive and the prevalence of TB co-infection was highest in sub-saharan Africa [48]. In the current study, TB was among the most frequent OIs especially in the period before HAART and prevalence was higher in men compared to women. The sex difference is consistent with many other previous studies which show men were more at risk of suffering from TB compared to women [50, 51]. The reason for this increased risk in men could probably be attributed to delayed enrolment on HAART by men compared to women [26]. In the current study it was also observed that TB was more frequent among HIV positive patients in Northern and Eastern Uganda compared to other geographical areas probably because of the socio-economic disparities in the regions. Previous studies show that poverty was a strong predictor of OIs even in the era of HAART [28, 52]. Generally TB prevalence was higher in older age groups (>30 years) which is consistent with other previous studies that shows TB prevalence increases with age [53]. A WHO TB prevalence survey 2014 in Indonesia found that TB prevalence was almost five times higher in older age groups (>40 years) compared to younger groups in both sexes [53].

Bacterial pneumonia (BP) caused by *Streptococcus pneumoniae* is one of the commonest respiratory tract infections in persons living with HIV/AIDS [4, 54–57]. A review of studies on HIV/AIDS related opportunistic infections in sub-Saharan Africa show high prevalence of *S. pneumonia* infection ranging from 25% in Cameroon to 31% in Uganda [4]. In our study, bacterial pneumonia was one of the commonly encountered opportunistic infection with an overall period prevalence of 14.8%. However its prevalence varied by geographical area with highest prevalence in Eastern Uganda. We also found BP was less in the period after HAART (2009–2013) at 8% compared to the period before HAART (13%) probably due to increased access to HAART and universal cotrimoxazole prophylaxis. Though a conjugate pneumococcal vaccine [54, 56] is now available in Uganda but it is still under limited access and we recommend that it should be availed to all HIV positive patients so as to reduce on the burden of bacterial pneumonia. It may be possible that bacterial pneumonia could have been under reported as in some cases, data were recorded as respiratory tract infection without specifying the infection.

In this study both AIDS defining and non-AIDS defining OIs were considered. Non-AIDS defining OIs found common among HIV positive patients in these settings were malaria and geohelminths. Given the geographical overlap of malaria and geohelminths with HIV in sub-Saharan Africa, there is great concern of the increasing number of helminthic and malaria co-infections among HIV positive patients [58–61]. Although malaria is not among the WHO defined opportunistic infections diagnostic of AIDS [62], several studies show that malaria tends to occur with increased frequency and severity in advanced HIV-infected adults [4, 63–68]. This happens probably because HIV infection reduces resistance to malaria by compromising the immune system. In our study we found malaria was very common among HIV positive patients especially before HAART consistent with previous studies elsewhere [4, 66–68]. According to the World Malaria Report 2014, Uganda was ranked 3rd after Democratic republic of Congo and Nigeria in contribution to the global burden of malaria [69]. Previous studies on malaria and HIV show that HIV increases vulnerability to malaria infection and malaria could enhance the progression of HIV infection to clinical AIDS in the absence of effective treatment [59, 70].

In the current study, prevalence of malaria men were less likely to suffer from malaria compared to women and the risk was less in those >35 years of age and varied by geographical area with the western Uganda having the highest risk compared to other geographical areas. Higher prevalence of malaria in younger age groups is consistent with previous findings that showed malaria prevalence to be inversely related with age [71–73]. Geographical variation in prevalence could be influenced by malaria endemicity in the different geographical areas.

Though malaria prevalence among HIV positive patients reduced in the era of HAART, it has not been completely eliminated. In view of the fact that malaria is highly endemic in Uganda and HIV positive patients are highly vulnerable, malaria prevention/control should therefore remain an integral part of comprehensive HIV/AIDS care in Uganda.

In the current study, geohelminths were the most commonly observed opportunistic infections among HIV positive patients before and after HAART. This is consistent with other studies elsewhere in resource poor settings. A study in Tanzania that investigated HIV and parasitic co-infections among HIV positive patients seeking care and treatment at health facilities in Tanzania found 22.1% had helminthic infections (hookworms, *Strongyloides stercoralis*, *Ascaris lumbricoides*, schistosomes), 12.9% had malaria and 13% had both [74]. A related study in Rwanda that investigated the prevalence of soil transmitted helminthes and malaria among HIV positive pregnant women attending antenatal health centers in Rwanda found 38% had helminthic infections (*A. lumbricoides*, *Trichuris trichiura*, *Ancylostoma duodenale* and *Necator americanus*), 21% had malaria and 10% had both [61]. Though geohelminths are not AIDS-defining opportunistic infections, previous studies show that co-infection with geohelminths was associated with dysregulation of the immune response causing inability of the HIV positive patient to mount an effective immune response [75]. High prevalence of geohelminths can lead to increased prevalence of anaemia thereby worsening the health conditions of persons living with HIV/AIDS [76]. Geohelminths have also been associated with diarrhea, nutritional impairment, abdominal pain and in children they can lead to impaired cognitive and physical development [58]. They have also been shown to accelerate the progression of HIV infection to AIDS [74, 77–80] and were found associated with increased risk for mother-to child-transmission of HIV [60].

Globally, it is estimated that about two billion people are infected with geohelminths mainly in resource limited settings [75]. These infections are however, often neglected in national programmes and yet they exact the greatest burden on limited resources in these settings [75]. They are commonly associated with settings characterised by poor sanitation and poor personal hygiene. Thus in most sub-Saharan Africa, the burden of helminthic infections is still enormous because of the poor sanitation and unhygienic conditions in these settings [81, 82].

However there were contradicting reports on the magnitude of helminthic infections among HIV/AIDS patients in resource poor settings. Some studies have shown higher prevalence of helminthic infections in HIV positive individuals compared to HIV negative controls [83, 84]. In contrast, others showed no significant difference in prevalence of helminthic infections between HIV positive patients and HIV negative controls [85, 86]. While others showed low prevalence of helminthic infections among HIV positive patients compared to HIV negative controls [87–89]. A number of studies have also shown that the patterns of individual helminthic parasites may differ among HIV positive and HIV negative patients. A study in Honduras found a strong association between *S. stercoralis* and HIV infection but lower risk for *A. lumbricoides* and *Trichuris trichurias* [90]. Another study found a high prevalence of *Strongyloidiasis* among HIV-positive patients compared to HIV negative controls in Brazil [91]. A related study in Ethiopia also found higher prevalence of *S. stercoralis* among HIV-positive patients with CD4 count <200 cells/µl [83].

In the current study, we were unable to assess this variation in intensity of infection by helminthic infection but we established that the prevalence of helminthic infections generally remained relatively higher compared to other opportunistic infections among HIV positive patients in Uganda even after HAART. However, more studies are required to have more insight on the role of HAART and age on severity of infection due to the different helminthic infections.

In the current study, it was also established that prevalence of geohelminths was lower among men compared to women and higher in older age groups (>35 years). We also observed variation in geographical distribution of geohelminths with Northern and Eastern Uganda having relatively higher burden of geohelminths than Central and Western Uganda. This variation in geographical distribution of geohelminths could be influenced by environmental and socio-economic factors including poverty, poor sanitation and personal hygiene, ignorance, lack of clean water and poor quality health care. In absence of vaccination, the recommended public health interventions would be regular deworming backed by access to clean water, improved sanitation and health education. A Cochrane systematic review of published literature on testing and treating HIV positive patients for intestinal helminthic infections showed that regular deworming with a single dose of albendazole is feasible in resource poor settings and would potentially improve survival and the quality of life of persons living with HIV/AIDs [75]. It is therefore important that regular deworming becomes an integral part of comprehensive HIV/AIDS care in Uganda and other countries in similar settings.

One of the limitations of this study was the fact that we used secondary data which had been collected not primarily for research purposes. Therefore, some variables which would have been useful in interpretation of our results, like patients’ viral loads, monitoring CD4 counts, BMI or ART adherence were missing for majority of the patients. These data should be targeted in prospective cohort studies in the future. Secondly, data from the four purposively selected HIV clinics used for this study may have not have been representative of all HIV positive individuals in Uganda which means generalisability could be limited to TASO programme in Uganda. Another limitation was that CD4 cell evaluations were not available for most patients and yet the study would have been be more informative if the rates of OI were reviewed in the context of CD4 cell counts. Thirdly, some OIs were not captured in the data base probably because of inadequate diagnostic capacity. Additionally, improvements in OI diagnosis like introduction of the lateral flow cryptococcal Antigen (CrAg) rapid tests for Cryptococcal meningitis and Gene Xpert for TB over time may have had an impact on prevalence of these OIs. Fourthly, cryptococcal meningitis and Kaposi’s prevalences could have been underestimated due to the fact they are referral conditions and the study clinics only handled out patients, so need to be interpreted with caution. Lastly the data presented doesn’t prove that HAART is responsible for the shift in OIs. However, we believe HAART greatly contributed to the reduction in the frequency of OIs, though it may not necessarily be the only factor. Other factors like general improvements in the quality of care and availability of more potent treatment drugs (fluconazole, etc.), antimicrobial prophylaxis and general improvement in socio-economic status over time could have also contributed to the overall reduction in the prevalence of OIs.

## Conclusions

Results from the current study show that the frequency and patterns of OIs have changed since the introduction of HAART in Uganda. However, these changes varied by type of OI, time period, age, gender and geographical location of the HIV positive patient. Geohelminths and diarrheal infection <1 month remains a challenge even after HAART and should therefore be given special attention in HIV/AIDS care programmes in these settings. Our findings further shows that opportunistic infections rather than opportunistic cancers were the primary cause of morbidity accounting for 99% of all the opportunistic episodes among HIV positive patients in these settings. However compared to other geographical areas in Uganda, HIV positive patients in Northern and Eastern Uganda bore the highest burden of OIs both before and after HAART and therefore should be given special consideration in terms of resource allocation and targeted interventions so as to reduce the disease burden due to OIs in these areas.

## Abbreviations

HAART: highly active antiretroviral therapy
HIV: human immunodeficiency virus
IQR: interquartile range
IRB: Institutional Review Board
AIDS: acquired immunodeficiency syndrome
OI: opportunistic infection
TASO: The AIDS Support Organisation
UNAIDS: Joint United Nations Programme on HIV/AIDS
WHO: World Health Organisation
SDG: sustainable development goal
